# Association between the triglyceride–glucose index and left ventricular global longitudinal strain in patients with coronary heart disease in Jilin Province, China: a cross-sectional study

**DOI:** 10.1186/s12933-023-02050-9

**Published:** 2023-11-22

**Authors:** Lin Na, Wenjing Cui, Xinqi Li, Jing Chang, Xin Xue

**Affiliations:** 1 Department of Cardiology, The Second Hospital of Jilin University, Changchun, China; 2 Department of Cardiology, Xi’an International Medical Center Hospital, Xi’an, China; 3 Clinical Laboratory, The Second Hospital of Jilin University, Changchun, China

**Keywords:** Coronary heart disease, TyG index, Myocardial strain, Cardiac function

## Abstract

**Background:**

This study aimed to investigate the association between the triglyceride–glucose (TyG) index and left ventricular global longitudinal strain (GLS) in patients with coronary heart disease and to examine the role of left ventricular GLS in detecting early changes in cardiac function in patients with coronary heart disease in the subclinical stage.

**Methods:**

A cross-sectional study involving 178 participants with symptomatic coronary artery disease excluding myocardial infarction or left ventricular dysfunction was conducted in Jilin Province, China. Basic clinical, biochemical, and echocardiographic data were obtained from all participants. Myocardial strain parameters were compared between patients with higher TyG index and those with lower TyG index, and the association between the gradually elevated TyG index and on subclinical cardiac function in patients with coronary heart disease was evaluated.

**Results:**

The GLS of left ventricle was lower in the higher TyG index group than in the lower TyG index group. As the TyG index increases, the GLS progressively decreases. The results remained stable after adjusting for confounding factors.

**Conclusions:**

A higher TyG index maybe independently associated with subclinical left ventricular dysfunction in patients with coronary heart disease.

**Supplementary Information:**

The online version contains supplementary material available at 10.1186/s12933-023-02050-9.

## Introduction

Coronary heart disease is the leading cause of death worldwide [1]. Patients with coronary heart disease can develop progressive heart failure despite revascularization. Heart failure is one of the most important complications of coronary heart disease [2] and is associated with high morbidity and hospitalization rates. However, in patients with coronary artery disease, adverse myocardial remodeling and myocardial dysfunction can occur years to decades before symptomatic heart failure owing to the presence of cardiovascular risk factors [3]. Insulin resistance (IR), a marker of metabolic disorders and systemic inflammation, has been shown to be strongly associated with the development of heart failure [3–12]. Recently, the triglyceride–glucose index (TyG index) [13], the product of the multiplication of fasting plasma glucose (FPG) and triglyceride (TG) levels, has been widely used in clinical practice as a simpler, less expensive, and more reliable index than the traditional Homeostatic Model Assessment of Insulin Resistance(HOMA-IR) index [14–18]. Existing studies have suggested that the TyG index may play an important role in the impairment of left ventricular structure and function [19–21]. Moreover, the TyG index has been shown to be associated with the exacerbation of heart failure in patients with coronary artery disease. However, in patients with symptomatic coronary artery disease excluding myocardial infarction or left ventricular dysfunction, whether the increase of the TyG index is associated with the early subclinical stage cardiac dysfunction is unknown and needs further investigation [22].

Although left ventricular ejection fraction (LVEF) is a commonly used parameter for evaluating left ventricular systolic function, it has low sensitivity for assessing local myocardial damage [23], does not accurately reflect the left ventricular myocardial function [24, 25], and cannot detect early myocardial dysfunction [26]. Myocardial strain has higher sensitivity and accuracy for evaluating cardiac function [27] and can assess the degree of myocardial deformation during the cardiac cycle [28], detect early changes in the left ventricular myocardium, and predict LVEF changes [29].

In this study, we used speckle-tracking technology to investigate the association between the TyG index level and left ventricular global longitudinal strain (GLS) in patients with coronary heart disease and to provide clinical evidence for the close association between the higher TyG index and the myocardial remodeling during subclinical stage.

## Methods

### Study population

This cross-sectional study was conducted in Jilin Province, China, between December 2021 and February 2022. A total of 178 patients with coronary heart disease were recruited from the Department of Cardiovascular Medicine at the Second Hospital of Jilin University. The diagnostic criterion for coronary heart disease was based on the American College of Cardiology and American Heart Association guidelines [30] (i.e., > 50% stenosis in at least one coronary artery confirmed with coronary angiography or computed tomography) [20]. Furthermore, all patients included in the study presented symptoms of angina pectoris and were admitted with varying degrees of symptom severity. The exclusion criteria included symptoms and signs of heart failure or ejection fraction ≤ 50%, arrhythmias affecting cardiac function, congenital or secondary cardiomyopathies, valvular and other structural heart diseases, and severe hepatic or renal insufficiency. Patients who had experienced coronary artery occlusion or previous acute myocardial infarction were excluded from undergoing coronary angiography or coronary CT. The eligible participants were divided into two groups according to the median TyG index value: TyG index < 8.96 group (n = 66) and TyG index > 8.96 group (n = 66).

All participants signed an informed consent form before their voluntary participation. This study was conducted in accordance with the guidelines of the Declaration of Helsinki and was approved by the ethics committee of the Second Hospital of Jilin University.

### Demographic and anthropometric characteristics

We recorded the sex, age, resting heart rate, and comorbidities, such as hypertension and hyperglycemia for each participant. Height and weight were measured twice, with the participants barefoot and in light clothing, and the average of the two values was calculated. Body mass index (BMI) was calculated by dividing the weight (kg) by the square of the height (m). Hypertension was defined according to the criteria of the World Health Organization [31]. Diabetes was diagnosed according to the criteria of the American Diabetes Association [32]. Hypertriglyceridemia was defined according to the Clinical Practice Guidelines on Hypertriglyceridemia from the Endocrine Society [33].

### Biochemical measurements

Blood samples were obtained from all participants, and laboratory tests were performed for the assessment of the estimated glomerular filtration rate (eGFR) and serum TG, serum high-density lipoprotein cholesterol, serum low-density lipoprotein cholesterol, serum total cholesterol (TC), fasting plasma glucose (FPG), and glycosylated hemoglobin levels.

### Baseline TyG index assessment

The TyG index was calculated as follows: Ln (fasting TG [mg/dL] × fasting glucose [mg/dL]/2). Blood samples for the measurement of plasma glucose and lipid levels were collected using a standard aseptic technique on the first day after patient admission. FPG levels were determined using the glucose oxidase method. TG levels were determined using an enzymatic method.

### Coronary Gensini scores

The Gensini score was calculated using coronary angiography findings [34]. On the basis of the segment with the most severe stenosis, a score of 1 point was assigned for stenosis diameter < 25%, 2 points for stenosis diameter between 25 and 49%, 4 points for stenosis diameter between 50 and 74%, 8 points for stenosis diameter between 75 and 89%, 16 points for stenosis diameter 90–98%, and 32 points for stenosis diameter ≥ 99%. The scores were multiplied by the corresponding coefficients for the coronary branches (left main branch, 5; proximal left anterior descending branch, 2.5; middle segment, 1.5; distal segment, 1; first diagonal branch, 1; second diagonal branch, 0.5; proximal left circumflex branch, 2.5; distal and posterior descending branches, 1; posterior collateral branch, 0.5; and proximal, middle, distal, or posterior descending branches of the right coronary artery, 1). The sum of the scores for all affected branches was the total score for each patient.

### Echocardiographic parameters

Before coronary angiography, echocardiographic examination was performed using an EPIQ 7C color Doppler ultrasound system (Philips, Amsterdam, Netherlands) with an X5-1 cardiac probe, at a probe frequency of 1.0–5.0 MHz. An experienced cardiac sonographer performed all measurements twice, in accordance with the American Society of Echocardiography criteria [35].

### Conventional echocardiography

Each cardiac parameter was assessed by calculating the average value from three consecutive cardiac cycles. All participants were in sinus rhythm at the time of echocardiographic examination. The Simpson biplane method was used to measure LVEF, left ventricular end-systolic volume, and left ventricular end-diastolic volume under hemodynamically stable conditions. Interventricular septal thickness was measured using M-mode ultrasonography. Pulsed-wave Doppler was used to measure the mitral valve E-peak (early diastole) and A-peak (late diastole) forward flow velocities, and the E/A ratio was calculated.

### Two-dimensional speckle-tracking echocardiography

All participants were instructed to hold their breath during image acquisition to obtain high-quality images. While acquiring the images, an electrocardiograph was used to track the left ventricular membrane from the parasternal long-axis and short-axis views and the apical two-chamber, three-chamber, and four-chamber views. Two-dimensional echocardiographic images of three to five complete cardiac cycles were obtained. The images were transferred to the Qlab13.0 workstation (Philips Healthcare, Andover, MA, USA) to automatically calculate the peak GLS of the left ventricle. The measurements were repeated three times for each participant, and the average was calculated. In accordance with the guidelines provided by the American Society of Echocardiography and European Society of Cardiovascular Imaging in 2016, for the measurement of cardiac chamber structures in adults [36], it is recommended that in all articles concerning strain changes, the terms referring to the increase or decrease in strain should be expressed as absolute values.

### Statistical analysis

According to the median value of the TyG index, patients with coronary heart disease were divided into two groups: TyG index < 8.96 group and TyG index > 8.96 group. Normally distributed data in the Kolmogorov–Smirnov test was expressed as mean ± standard deviation, and the t-test was used for comparisons between two groups. Data with skewed distributions were expressed as median (interquartile range), and the rank sum test was used to compare two groups. Count data were expressed as number (n) and percentage (%) and compared using the chi-square test. Pearson’s or Spearman’s correlation analyses were used to assess correlations between left ventricular GLS and cardiometabolic risk factors.

We assessed the association between the TyG index and the risk of GLS using a multivariate linear regression model by calculating effect sizes (β) and 95% confidence intervals (CIs). Five models were used to adjust for potential confounders: model 1 was the crude model; model 2 was adjusted for age and sex; model 3 was further adjusted for comorbidities, including hypertension and diabetes; model 4 was further adjusted for risk factors with *P* values of < 0.05 in univariate analysis or variables that, when added to this model, changed the matched β by at least 10%; and model 5 was adjusted for the clinically meaningful Gensini scores. Multicollinearity between covariates was assessed using the variance inflation factor and tolerance values (Additional file 1). Statistical significance was set at *P* < 0.05. In these models, the median of the TyG-index quartiles was used as the continuous variable in the regression model to conduct linear trend tests. Additionally, curve fitting was used to evaluate the linear dose–response association between baseline TyG index and GLS.

To assess the robustness of the association, we performed a pre-specified subgroup analysis. In the subgroup analysis, we examined the relationship between GLS impairment and the TyG index according to age (≤ 65 years vs. > 65 years), sex (male vs. female), hypertension (no vs. yes), diabetes (no vs. yes), and hypertriglyceridemia (no vs. yes). Interactions between subgroups were assessed using the likelihood ratio test.

All statistical analyses were performed using R version 3.3.2 (http://www.R-project.org; The R Foundation for Statistical Computing, Vienna, Austria), Free Statistics software version 1.7.1, and SPSS Statistics version 25.0 (IBM Corp., Armonk, NY, USA). The central illustration were created with permission in BioRender.com (https://biorender.com, accessed on 30 June 2023). All analyses were performed using a two-tailed test, and *P* < 0.05 was considered statistically significant. The sample size was calculated using PASS version 11 (NCSS Statistical Software, Kaysville, UT, USA). On the basis of the pre-test results, the average GLS in the low TyG index group was 17.5 ± 3.7% and that in the high TyG index group was 14.2 ± 4.0%. For a two-sided α of 0.05, power of 95%, and a ratio between the two groups of 1:1, at least 25 participants were required in each group.

## Results

**Fig. 1:**
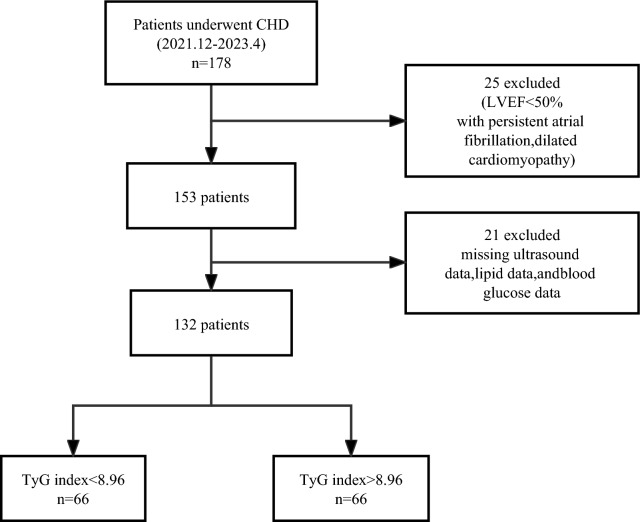
Flowchart of participant selection

Of the initially evaluated 178 patients with coronary heart disease, 46 were excluded (16 had an ejection fraction of < 50%, 8 had missing ultrasound data, 6 had persistent atrial fibrillation, 3 had dilated cardiomyopathy, 7 had missing lipid data, and 6 had missing blood glucose data). Therefore, 132 patients met the inclusion criteria. Figure 1 shows the flowchart of the patient selection process.

The 132 participants (male proportion, 59.8%) had a mean age of 62.9 years. Of these participants, 66 had a TyG index of ≥ 8.96 and 66 had a TyG index of < 8.96. We used single regression imputation to estimate missing values for the covariates (missing values < 10%). Table 1 shows the basic clinical characteristics of the study population grouped according to the TyG index. Patients in the TyG index ≥ 8.96 group had a higher resting heart rate (*P* = 0.037) and a higher prevalence of diabetes mellitus (*P* = 0.001) than patients in the TyG index < 8.96 group. At the same time, there were more female patients in the high TyG index level group. No significant differences were observed in age, BMI, or prevalence of hypertension between the two groups (*P* > 0.05). In terms of biochemical indicators, TG, TC, glycosylated hemoglobin, FPG, and the TyG index were significantly higher in the TyG index ≥ 8.96 group than in the TyG index < 8.96 group (*P* < 0.01). The high-density lipoprotein level was lower in the hypertriglyceridemia group than in the non-hypertriglyceridemia group, and the difference was statistically significant (*P* = 0.005). No significant differences in low-density lipoprotein cholesterol levels, eGFR and the application of medication were observed between the two groups (*P* > 0.05). In addition, the Gensini scores were not significantly different between the two groups (*P* > 0.05).

**Table 1:** Basic clinical characteristics of the participants

Variables	Total (n = 132)	0 (n = 66)	1 (n = 66)	p
Age, Mean ± SD	62.9 ± 9.8	63.8 ± 10.4	62.9 ± 9.1	0.309
Sex (M %)	75(56.8%)	46(69.7%)	29 (43.9%)	0.003
HR	76.1 ± 10.6	74.2 ± 9.5	78.0 ± 11.3	0.037
BMI	26.0 ± 4.1	25.4 ± 3.9	26.5 ± 4.4	0.124
Hb	140.5 ± 17.8	141.7 ± 16.0	139.3 ± 19.4	0.456
Hypertension n (%)	82(63.1%)	38(58.5%)	44(67.7%)	0.276
Diabetes mellitus (%)	50 (37.9)	16 (24.2)	34 (51.5)	0.001
eGFR (ml/min/1.73m2)	87.2 (74.0, 99.0)	87.1 (73.0, 99.2)	87.4 (76.4, 98.7)	0.822
TC (mmol/l)	4.0(3.3,5.0)	3.8 (3.0, 4.6)	4.4 (3.6, 5.4)	< 0.001
TG (mmol/l)	1.6 (1.1, 2.2)	1.2 (0.9, 1.4)	2.2 (1.7, 3.2)	< 0.001
HDL.C (mmol/l)	0.9(0.8,1.1)	1.0(0.9,1.2)	0.9(0.8,1.0)	0.005
LDL.C (mmol/l)	2.5(1.8,3.2)	2.5 (1.7, 3.1)	2.5 (1.9, 3.2)	0.296
HbA1c (%)	6.1(5.7,7.4)	5.9(5.7,6.3)	6.9 (5.7, 8.3)	< 0.001
Fasting glucose (mmol/l)	5.9 (5.0, 7.4)	5.2 (4.8, 5.9)	7.0 (5.5, 9.2)	< 0.001
TyG index	9.0 (8.6, 9.4)	8.6 (8.3, 8.8)	9.4 (9.1, 9.8)	< 0.001
GENSINI score	37.0 (23.8, 66.5)	36.5 (21.0, 59.5)	37.5 (25.2, 72.8)	0.340
ACEI/ARB, n (%)	29 (22.0)	14 (21.2)	15 (22.7)	0.833
CCB, n (%)	43 (32.6)	18 (27.3)	25 (37.9)	0.194
Statin, n (%)	0 ( 0.0)	0(0)	0 (0)	1
oral hypoglycemic drugs	17 (12.9)	6 (9.1)	11 (16.7)	0.194
Insulin, n (%)	36 (27.3)	13 (19.7)	23 (34.8)	0.051

TyG: triglyceride–glucose; SD: standard deviation; HR: heart rate; BMI: body mass index; Hb: hemoglobin; eGFR: estimated glomerular filtration rate; TC: total cholesterol; TG: triglyceride; HDL-C: high-density lipoprotein cholesterol; LDL-C: low-density lipoprotein cholesterol; HbA1c: glycosylated hemoglobin; FPG: fasting plasma glucose

**Fig. 2:**
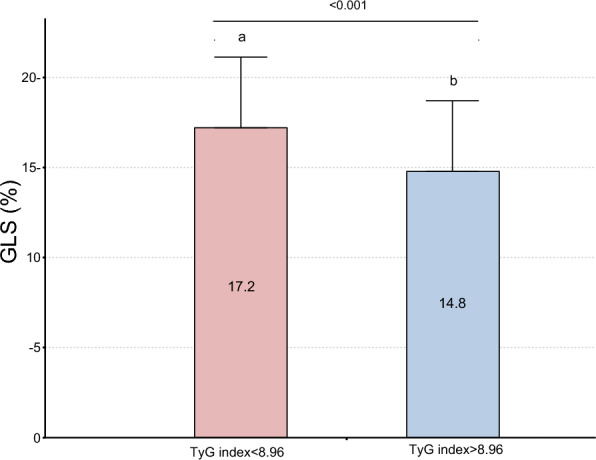
Comparison of GLS between high TyG index levels and control groups

Table 2 shows the echocardiographic characteristics of the two groups. The routine echocardiographic parameters showed no statistically significant differences between the two groups. The GLS was significantly impaired in the TyG index ≥ 8.96 group compared with that in the TyG index < 8.96 group (*P* < 0.05, Fig. 2).

**Table 2:** Echocardiographic characteristics of all participants

Variables	Total (n = 132)	TyG index < 8.96 (n = 66)	TyG index > 8.96 (n = 66)	p
Routine				
LVEF	62.6 ± 5.8	62.1 ± 5.0	63.0 ± 6.5	0.393
LVESV	50.9 (36.3,61.2)	49.5 (36.2,59.3)	50.9 (36.8,61.9)	0.626
LVEDV	130.0 (104.0, 151.0)	127.0(100.0,149.0)	137.8 (105.8,155.0)	0.308
E	64.0 (54.0, 77.1)	64.3 (52.1, 76.4)	62.6 (54.8, 78.1)	0.935
A	91.8 ± 15.9	90.2 ± 16.6	93.4 ± 15.1	0.250
E/A	0.7(0.6,0.8)	0.7(0.6,0.8)	0.7(0.6,0.8)	0.795
Myocardial strain				
GLS	16.0 ± 4.1	17.2 ± 3.9	14.8 ± 3.9	< 0.001

TyG: triglyceride–glucose; LVEF: left ventricular ejection fraction; LVESV: left ventricular end-systolic volume; LVEDV: left ventricular end-diastolic volume; E: early diastolic mitral valve peak E velocity; A: peak late mitral inflow velocity; GLS: global longitudinal strain

Table 3 shows the correlations between potential risk factors and GLS. Correlation analysis showed that the TyG index, TG, TC, FPG, resting heart rate, and eGFR were correlated with GLS in patients with coronary heart disease. Among these factors, the TyG index was most closely related to GLS, showing a moderate correlation (r = 0.422, *P* < 0.05). TG, as a component of the TyG index, also showed a moderate correlation with GLS (r = 0.371, *P* < 0.05). Resting heart rate (r = 0.289, *P* = 0.001), TC (r = 0.209, *P* = 0.017), FPG (r = 0.242, *P* = 0.005), eGFR (r =  − 0.185, *P* = 0.034) and GENSINI score (r = r = 0.223, P = 0.010) were weakly correlated with GLS.

**Table 3:** Correlation analysis of potential risk factors for GLS

Parameter	r	P
Age (years)	0.039	0.658
BMI (kg/m^2^)	− 0.062	0.483
HR (beat/min)	0.289	0.001
Triglycerides (mmol/L)	0.371	< 0.0001
Total cholesterol (mmol/L)	0.209	0.017
HDL-C (mmol/L)	− 0.172	0.049
LDL-C (mmol/L)	0.077	0.380
Fasting glucose (mg/dl)	0.242	0.005
HbA1c (%)	0.155	0.078
TyG index	0.422	< 0.0001
eGFR (ml/min/1.73m^2^)	− 0.185	0.034
Gensini score	0.223	0.010

**Fig. 3:**
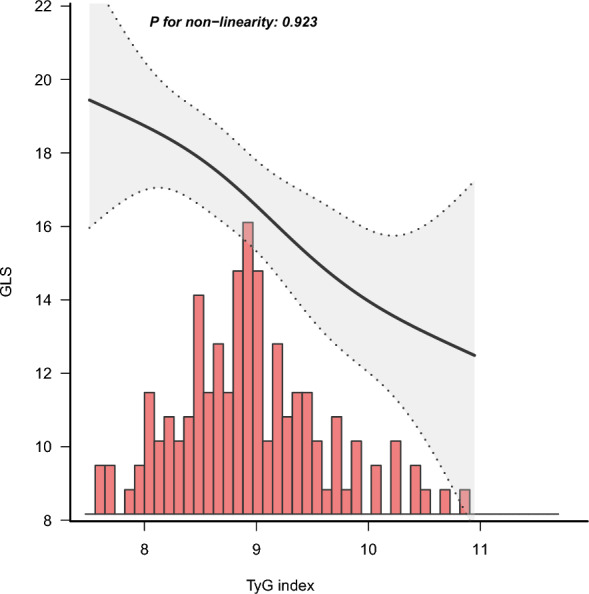
The fitting curve between the TyG index level and GLS

Figure 3 shows a fitting curve between the TyG index level and GLS. To evaluate the dose–response relationship between the TyG index and GLS in patients with coronary heart disease after adjusted for model 5 covariates, we constructed a fitting curve. As shown in the Fig. 3, there is a negative linear relationship between the TyG index level and GLS in patients with coronary heart disease.

**Fig. 4.**
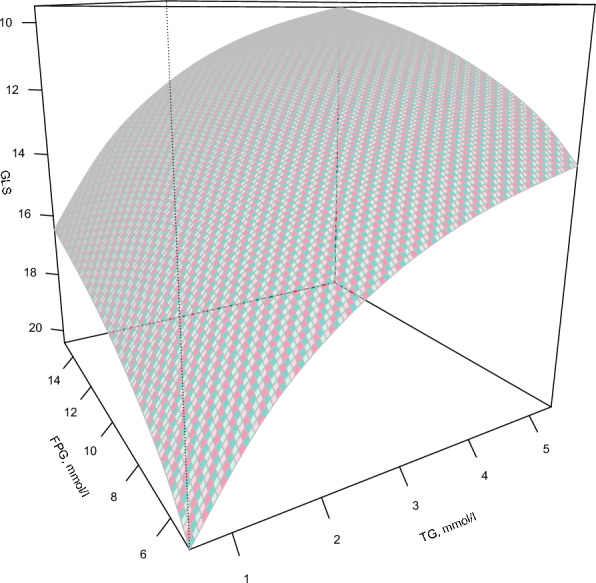
3-Dimensional mesh plot of the interplay between TG, FPG, and GLS in unit

Figure 4 shows the three-dimensional mesh plot illustrating the relationship between fasting glucose, triglycerides, and the GLS. It can be concluded from the figure that as triglyceride and fasting blood glucose levels increase, the value of GLS shows a gradually decreasing trend, indicating that GLS is gradually being damaged. The lowest value of GLS is observed when both triglyceride and fasting blood glucose levels are high.

To further investigate whether the TyG index maybe independently associated with left ventricular GLS in patients with coronary heart disease, we constructed a multivariate linear regression model (Table 4). Both non-adjusted and multivariate-adjusted models were used to verify the stability of the results. Variables for adjustment were selected on the basis of the following three criteria: (1) variables that, when added to the model, would change the matched β by at least 10%; (2) variables with *P* < 0.05 in the univariate linear regression analysis; or (3) variables that were considered confounders based on existing literature and clinical judgment. Variables with strong multicollinearity were excluded. Based on the results of the tolerance value and variance inflation factor tests, the variables in the final model did not have multicollinearity. Adjusting for confounding factors did not change the association between the TyG index and GLS (β = − 2.015, 95% CI − 0.919 ~ − 3.111 P < 0.001). For every 1 unit increase in the TyG index, the GLS decreased by 2.015-unit, indicating that a high TyG index level maybe independently associated with subclinical left ventricular dysfunction in coronary heart disease patients.

**Table 4:** multivariate analysis of parameters associated between TyG index and GLS

Model	P	β	95% CI
Model1	< 0.001	− 2.504	− 3.411 to − 1.597
Model2	< 0.001	− 2.715	− 3.658 to − 1.773
Model3	< 0.001	− 2.625	− 3.591 to − 1.659
Model4	< 0.001	− 2.046	− 3.153 to − 0.940
Model5	< 0.001	− 2.015	− 3.111 to − 0.919

Model 1: crude model

Model 2: adjusted for age and sex

Model 3: adjusted for model 2 covariates + hypertension and diabetes

Model 4: adjusted for model 3 covariates + heart rate, estimated glomerular filtration rate, and total cholesterol

Model 5: adjusted for model 4 covariates + Gensini score

P < 0.05 indicates significance

Table 5 presents the trend test conducted between the TyG index and GLS. As part of the sensitivity analysis, we categorized the TyG index into quartiles and included them as variables in the regression model, and set the highest quartile group of TyG index as the reference group. The findings indicate that there is a significant linear trend between the TyG index and GLS, with a P-value for trend < 0.05. As the quartile level of the TyG index increases, there is a gradual decrease in the value of GLS, suggesting a decline in left ventricular myocardial systolic function.

**Table 5:** Trend test of changes in TyG index and GLS

Model	Quartile of TyG index				
	Q1 (< 8.56)	Q2 (8.56–8.96)	Q3 (8.96–9.34)	Q4 (> 9.34)	
	β (95CI%)	β (95CI%)	β (95CI%)	β (95CI%)	P for trend
Model1	4.12 (2.29–5.96)	3.76 (1.93–5.59)	3.03 (1.20–4.87)	0 (Reference)	< 0.001
Model2	4.56 (2.60–6.53)	3.79 (1.91–5.67)	3.03 (1.18–4.87)	0 (Reference)	< 0.001
Model3	4.31 (2.29–6.33)	3.62 (1.70–5.55)	2.89 (1.02–4.77)	0 (Reference)	< 0.001
Model4	3.20 (1.03–5.37)	2.97 (1.03–4.90)	2.24(0.35–4.13)	0 (Reference)	0.003
Model5	3.06 (0.91–5.21)	3.06 (1.15–4.98)	2.07(0.19–0.19)	0 (Reference)	0.003

Model 1: crude model

Model 2: adjusted for age and sex

Model 3: adjusted for model 2 covariates + hypertension and diabetes

Model 4: adjusted for model 3 covariates + heart rate, estimated glomerular filtration rate, and total cholesterol

Model 5: adjusted for model 4 covariates + Gensini score

P < 0.05 indicates significance

**Fig. 5:**
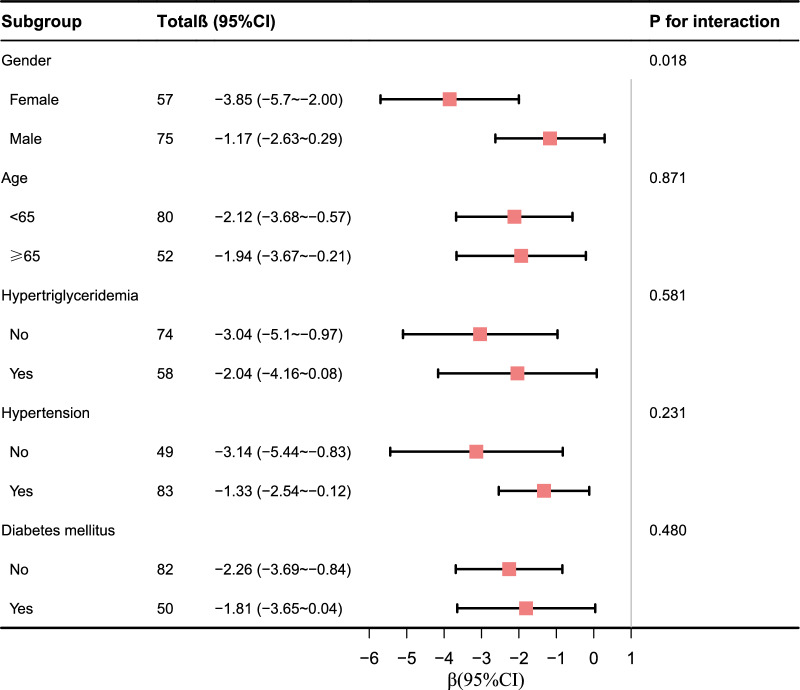
Stratification analysis on the association between TyG index and GLS in coronary heart disease

A forest plot of the subgroup analysis is shown in Fig. 5. Subgroup analysis showed a significant interaction between sex and the TyG index (*P* for interaction = 0.018). The association between the TyG index and GLS was more significant in female patients than in male patients (β − 3.85 [95% CI − 5.7 ~  − 2.00] for female patients vs. β − 1.17 [95% CI − 2.63 ~ 0.29] for male patients). Subgroup analysis showed that the association between the TyG index and GLS similar across patient subgroups stratified according to age, sex, hypertension, diabetes mellitus and hypertriglyceridemia (*P* values for interaction > 0.05).

## Discussion

In this study, we found that with an increase in the TyG index in patients with coronary heart disease, the value of GLS decreased. The correlation existed after adjusting for the effects of confounding factors (e.g., age, sex, comorbidities, TC, resting heart rate, Gensini score, and eGFR) in multivariate linear regression analysis, indicating that the increase of TyG index may be independently associated with subclinical left ventricular dysfunction in coronary heart disease patients. We also found that the increase in the TyG index was associated with more significant in subclinical left ventricular dysfunction in female patients than in male patients with coronary heart disease.

Previous studies on increased TyG index and its impact on cardiac function mostly focused on patients with non-coronary heart disease and on the occurrence of heart failure events. Our results are consistent with those of other studies on different patient groups. Liu et al. utilized the Third National Health and Nutrition Examination Survey database and found that an increased TyG index was independently associated with the risk of subclinical myocardial injury in a population of American patients without a history of coronary heart disease [37]. Huang et al. found that a high TyG index was significantly associated with a higher risk of heart failure and impaired left ventricular structure in a US community population without heart failure or coronary heart disease, and that a larger baseline TyG index was associated with unfavorable left ventricular remodeling and left ventricular dysfunction [19]. Furthermore, some studies have found that a higher TyG index can predict the occurrence of congestive heart failure in young adults in the United States [38]. However, the results from both a prospective study performed in Kailuan, China, and a retrospective cohort study performed in Hong Kong, China, suggest a possible causal relationship between a higher TyG index and an increased risk of heart failure in persons without cardiovascular disease [39]. A study by Xu et al. also demonstrated that the TyG index was positively associated with the risk of new heart failure events and that the TyG index may help identify high-risk groups for developing heart failure [40]. In adults with essential hypertension, the TyG index has been shown to be a potential marker for the development of heart failure [22]. Additionally, a previous study demonstrated that the TyG index was higher in patients with essential hypertension and heart failure with preserved ejection fraction (HFpEF) [41] than in patients without HFpEF. In another study, a high TyG index was associated with left ventricular diastolic dysfunction and structural abnormalities in patients with newly diagnosed hypertension [42]. Some scholars have discovered that an increased TyG index in patients with diabetes is closely related to cardiac hemodynamics, and it is expected to become a biological indicator for predicting changes in cardiac function in those with type 2 diabetes mellitus [43]. Two separate cross-sectional studies conducted in China on diabetic patients without coronary heart disease have demonstrated an independent association between the TyG index and subclinical left ventricular dysfunction in patients with type 2 diabetes [44, 45]

Currently research data on the association between the TyG index and early myocardial dysfunction in the subclinical stage in patients with coronary heart disease are still lacking, and no relevant study has been published. The present study fills these gaps and highlights the GLS decreases with increasing TyG index, which has important clinical implications. In addition, our study is more suitable for the population of Northeast China. To our knowledge, this is the first clinical study on the relationship between the TyG index and GLS in patients with coronary heart disease in Northeast China. Additionally, this study is innovative because it used myocardial strain technology to evaluate outcome indicators. The results of our study may be helpful for the early prevention of cardiac function damage in patients with coronary heart disease during the subclinical stage of left ventricular dysfunction.

To date, no definite conclusion has been drawn regarding the mechanisms underlying the association between elevated TyG index and subclinical left ventricular dysfunction in patients with coronary heart disease [9, 46]. Previous studies have shown a positive correlation between an increase in the TyG index and the process of myocardial fibrosis [47, 48]. The mechanisms that affect myocardial structural changes can be divided into several categories, as described below.

The TyG index plays a role in the occurrence of myocardial structural changes by affecting glucose and lipid metabolism. As a composite index of TG and FPG, the TyG index is closely related to IR [49] and metabolic syndrome [49–51]. An increase in the TyG index leads to myocardial lipotoxic injury. Additionally, an increased TyG index indicates systemic aggravation of IR. Elevated insulin levels stimulate the transport of free fatty acids (FFA) into cardiomyocytes [52]. If the FFA influx exceeds the oxidative capacity of the cells, large amounts of lipids and intermediates will accumulate in the myocardium [53]. As a result, the epicardial fat pad expands, which has direct toxic effects on the subendocardial cavity and the myocardium (i.e., “lipotoxicity”) [54]. This eventually leads to myocardial fibrosis and myocardial contractile dysfunction. In contrast, an increased TyG index affects the myocardial structure by interfering with glucose metabolism. IR leads to chronic systemic hyperglycemia. Glucotoxicity can exacerbate cardiac injury through the direct and indirect effects of glucose on cardiomyocytes, cardiac fibroblasts, and endothelial cells [55]. High levels of blood glucose also alter the cardiac structure and function through post-translational modifications of extracellular matrix proteins and altered expression or function of the calcium channels in cardiomyocytes, possibly promoting myocardial systolic and diastolic dysfunction [56].

In addition, exacerbated insulin resistance leads to increased activity of the sympathetic nervous system and the renin–angiotensin–aldosterone system [46, 57, 58]. Chronic hyperinsulinemia can lead to increased angiotensinogen production and upregulated expression of the angiotensin II receptor in adipose tissue [59], further promoting the generation of ROS in tissues such as the myocardium [60]. This process is possibly mediated by inflammation, cardiac fibrosis, and oxidative stress, which promote cardiac remodeling [61–64].

A high TyG index can act on the vasculature and promote coronary artery stenosis [4], leading to heart failure by increasing the risk of myocardial ischemic injury [65–67]. Specifically, chronic inflammation caused by IR [68–70] and endothelial dysfunction may contribute to vulnerable plaque formation [66, 71, 72], impairing the compensatory mechanisms of the myocardium and making it susceptible to ischemia and pressure overload [73]. Consequently, in-stent restenosis occurs after PCI in patients with coronary heart disease, which aggravates ischemic myocardial cell injury. Obstructed coronary arteries promote myocardial fibrosis, which further affects the left ventricular myocardial function.

In sex-stratified subgroups in the present study, the association between the TyG index and the risk of GLS was stronger in female patients than in male patients with coronary heart disease. The difference in the risk of myocardial injury caused by this sex difference has also been reported in other studies. A study by Zou and Xu in patients who underwent PCI and drug-eluting stent implantation showed that an increase in the TyG index in female patients was significantly related to the occurrence of major adverse cardiovascular and cerebrovascular events; however, this relationship was not observed in male patients [74]. Similarly, the results of a clinical study by Li showed that, in patients without prevalent cardiovascular disease, the association between the TyG index and the heart failure risk was more significant in female patients than in male patients. The mechanisms responsible for this difference are unclear and may be derived from differences in hormonal axes between the sexes. Different sex hormones further affect the myocardial structure and function by acting on lipid and glucose metabolism [75]. Previous studies have reported sex differences in TG metabolism [76, 77]. Owing to the high correlation between TG levels and the TyG index, the association between the TyG index and GLS may differ between male and female patients with coronary heart disease.

## Study limitations

This study had some limitations. First, as this was a cross-sectional study with a small sample size, no causal relationships could be established. Unmeasured potential confounding factors may exist when assessing the association between the TyG index and the GLS of the left ventricle in patients with coronary heart disease; however, the results of our sensitivity analysis showed that the results were stable across different subgroups. Second, left ventricular global circumferential strain and global radial strain were not assessed. Nevertheless, the GLS of the left ventricle is more readily available in clinical practice than the left ventricular global circumferential strain and global radial strain and is useful for detecting subclinical systolic dysfunction in high-risk patients. In future studies, stratified strain analysis should be considered and left ventricular diastolic function should be further evaluated. Third, this study used two-dimensional speckle-tracking technology to obtain ultrasound images. With this technology, the image quality is limited by the conditions of the acoustic window, such that low-echo areas may be difficult to view.

In summary, in patients with coronary heart disease, a high TyG index maybe independently associated with subclinical left ventricular dysfunction in coronary heart disease patients. Attention should be paid to maintaining the optimal glycolipid levels, as it can delay the progress of clinical heart failure in patients with coronary heart disease.

### Supplementary Information

**Additional file 1.** Co-linearity analysis between covariates.

## Data Availability

The datasets generated and/or analysed during the current study are not publicly available due data from this study may contain potentially or sensitive patient information, but are available from the corresponding author on reasonable request.
