# Supplementary material for: Association between the triglyceride–glucose index and left ventricular global longitudinal strain in patients with coronary heart disease in Jilin Province, China: a cross-sectional study
Source: Cardiovasc Diabetol. 2023 Nov 22;22:321. doi: 10.1186/s12933-023-02050-9 (PMC10666388; doi:10.1186/s12933-023-02050-9)
Supplement: Supplementary file 1 — Additional file 1. Co-linearity analysis between covariates. [file 12933_2023_2050_MOESM1_ESM.docx]

Supplementary Material. Co-linearity analysis between covariates.

|  | | Unstandardized  coefficients | | Standardized  coefficients | t | Sig. | Collinearity  statistics | |
| --- | --- | --- | --- | --- | --- | --- | --- | --- |
|  |  | B | Std. error | Beta |  |  | Tolerance | VIF |
|  | (Constant) | 23.232 | 4.711 |  | 4.932 | <0.001 |  |  |
|  | Diabetes mellitus | -0.998 | 0.725 | -0.119 | -1.377 | 0.171 | 0.856 | 1.168 |
|  | Hypertension | 1.025 | 0.697 | 0.122 | 1.471 | 0.144 | 0.933 | 1.071 |
|  | Age | -0.007 | 0.039 | -0.016 | -0.168 | 0.867 | 0.735 | 1.360 |
|  | Sex | 0.013 | 0.697 | 0.002 | 0.019 | 0.985 | 0.901 | 1.110 |
|  | HR (beat/min) | -0.101 | 0.032 | -0.260 | -3.121 | 0.002 | 0.929 | 1.077 |
|  | TC(mmol/l) | -0.379 | 0.243 | -0.133 | -1.563 | 0.121 | 0.888 | 1.126 |
|  | GENSINI score | -0.017 | 0.008 | -0.171 | -2.012 | 0.046 | 0.888 | 1.126 |
|  | Egfr(ml/min/1.73m^2^) | 0.036 | 0.019 | 0.175 | 1.879 | 0.063 | 0.743 | 1.346 |

Dependent variable：GLS.

Co-linearity analysis showed there is no high co-linearity between covariates.
